# Verifying the Use of Food Labeling Data for Compiling Branded Food Databases: A Case Study of Sugars in Beverages

**DOI:** 10.3389/fnut.2022.794468

**Published:** 2022-02-03

**Authors:** Edvina Hafner, Živa Lavriša, Maša Hribar, Sanja Krušič, Anita Kušar, Katja Žmitek, Mihaela Skrt, Nataša Poklar Ulrih, Igor Pravst

**Affiliations:** ^1^Nutrition and Public Health Research Group, Nutrition Institute, Ljubljana, Slovenia; ^2^Biotechnical Faculty, University of Ljubljana, Ljubljana, Slovenia; ^3^VIST–Faculty of Applied Sciences, Ljubljana, Slovenia

**Keywords:** beverages, reformulation, added sugar, food composition, HPLC, database

## Abstract

Branded food composition databases are an important tool for research, education, healthcare, and policy making, amongst others. Such databases are typically compiled using food labeling data without chemical analyses of specific products. This study aimed to verify whether the labeled sugar content in sugar-sweetened beverages (SSBs) corresponds to the actual sugar content in these products, thus enabling food monitoring studies to be conducted. A secondary objective was to determine the specific types of sugars in these SSBs. A case study was conducted using market share-driven sampling of these beverages from the Slovenian food supply. On the basis of nationwide yearly sales data, 51 best-selling products were sampled in 2020 and analyzed using high-performance liquid chromatography. This sales-driven approach to sampling has been shown to be very useful for conducting food monitoring studies. With the careful selection of a small proportion of available products, we finished with a manageable sample size, reflecting the composition of a majority (69%) of the national market share volume. The analyzed total sugar content was compared with labeled data, within the context of the European Union's regulatory labeling tolerances. In all samples, the sugar content was within the tolerance levels. The most common (*N* = 41) deviation was within ±10% of the labeled sugar content. In the subcategories, the differences between the analyzed and labeled median sugar contents were not statistically significant. Sucrose was most commonly (*N* = 36; 71%) used for sweetening, suggesting that the proportion of fructose in most SSBs was around 50%. A higher fructose content was only observed in beverages with fructose–glucose syrup or a higher content of fruit juice. The study results show that the labeled sugar content information in SSBs is reliable and can be used to compile branded food databases and monitor the nutritional quality of foods in the food supply.

## Introduction

Food composition databases are an important tool for research, education, healthcare, and policy making, amongst others (1). Epidemiological nutritional studies mostly convert food consumption data into nutrient intake data based on food composition databases (2, 3). While databases for generic foods are typically built based on complex laboratory analyses, such datasets lack details of the nutritional composition of specific processed (branded) foods (4). Because branded foods are gaining importance in people's diets, branded food composition datasets have become a common data source used in nutrition research (5, 6). The standard methodology for compiling branded food databases is cross-sectional data collection in food stores, where information is extracted from food labels (7–9). Trustworthy information on branded foods can improve the accuracy of nutrient intake data, support the monitoring of food reformulation progress, and empower consumers to make informed food choices.

Data in branded food databases, however, are often limited in terms of availability and accuracy due to the information provided by manufacturers on the food labels. In the European Union (EU), food labeling is regulated by Regulation (EU) 1169/2011, i.e., certain food information must be provided to the consumer (10). This mandatory nutritional declaration typically consists of energy value and contents of fats, saturated fats, carbohydrates, sugar, protein, and salt; the provision of other information such as fiber, vitamins, and minerals is optional. According to the regulation, food manufacturers are responsible for aligning the information provided in the nutritional declaration with the actual composition of the food. The regulation also allows manufacturers to either provide the results of laboratory analyses or to estimate the nutrient contents based on the known composition of the ingredients used in the production process (11). While laboratory analyses using valid procedures are the optimal choice for this purpose, these often involve significant costs and are therefore not commonly performed. Because the nutritional composition of foods is affected by various factors, including production and storage, often products do not contain the exact nutrient values stated on their labels. For this reason, the European Commission (EC) issued guidance in order to establish tolerances for specific nutrients in the nutritional declaration so that labeled and actual contents do not substantially differ (12).

In EU countries, the regulatory control of food labeling depends on local laws and each country's law enforcement bodies; i.e., each country implements their own enforcement rules and fines as they relate to Regulation (EU) 1169/2011 (10). To support harmonized food control, the EC issued guidance on how the responsible authorities should address compliance with the EU legislation (12). Food control plans of these authorities include the verification of nutrient values declared on a label. While all countries are encouraged to share these verification reports with the EC, to the best of our knowledge, such reports are not readily available to the public. The authority responsible for this food control function in Slovenia is the Administration for Food Safety, Veterinary Sector and Plant Protection and Health Inspectorate of the Republic of Slovenia. Penalties for violating the Regulation (EU) No. 1169/2011 vary from country to country and depend on the type of violation. For example, in Slovenia, the penalty for providing inadequate nutritional value information ranges from 500 € to 15,000 € (e.g., for foods not labeled with nutritional information or that provide inaccurate health claims) (13), while in Italy, penalties for violating Regulation (EU) No. 1169/2011 are in a range from 500 € to 40,000 € (14).

Although food control in Europe is carefully regulated, the process is mostly focused on areas with pre-identified food safety risks, making the information provided in the nutritional declaration a low priority for the authority responsible (15). As food manufacturers are responsible for the accuracy of the information on food labels, questions frequently arise as to whether this information is sufficiently accurate and suitable for compiling branded food databases and for use in nutrition research. Very few studies have compared the actual (analyzed) nutritional composition of foods with the nutrient information provided on food labels, and the two sets of information are sometimes contradictory. For example, in 2011, a study from the United States (US) showed notable differences between labeled and analyzed sugar contents in soft drinks (16). Similarly, in Malaysia, they found that only 66% of the products complied with their legislative limits as required for the nutritional declaration (17). In Europe, studies have shown better compliance with EU tolerance ranges, as stated in Regulation (EU) 1169/2011 (10), but the results still varied. In Portugal, the highest compliance was found for fats (88%), while it was lower for salt (74%) and saturated fats (73%) (18). A study in Ireland found that labeled sugar contents in yogurts was among the least reliable information on the label (19). Much better compliance was demonstrated in a recent Spanish study (20), where 98.4% of the analyzed processed (branded) foods met the EU tolerance range for sugars. A key challenge in all of these studies is sample selection, since there are thousands of foods on the market and sampling/analysis capacities are typically much more limited. While a common randomized sample selection is a common approach, sampling driven by market share is a more practical and relevant approach, i.e., it better reflects the overall food supply.

Sugar-sweetened beverages (SSBs) are one of the key food categories addressed in public health policies (21). SSBs have been shown to affect oral health, weight gain, and increase the risk of chronic conditions such as obesity, diabetes, fatty liver disease, and cardiovascular diseases (21–25). The main health risk of SSBs is that they contain high amounts of free sugar and are consumed in large quantities by vulnerable population groups. Although the World Health Organization (WHO) recommends that dietary intake of free sugars should be <10% of a person's daily energy intake (26, 27), a large proportion of Europeans easily exceed this intake limit. For example, SSBs have been identified as a major contributor to free sugar intake among several population groups, particularly children/adolescents (2, 28, 29). The results of several epidemiological studies suggest that sugars in beverages can lead to a greater risk of developing metabolic syndrome than sugar in other foods. A plausible reason for this observation is that fructose and its unique associated metabolism have specific negative health effects (30, 31). SSBs often have a higher fructose content, which is better absorbed by the body and results in higher concentrations stored in the liver (32). Higher amounts of fructose are often found in SSBs because manufacturers increasingly use concentrated fruit juice and fructose–glucose syrup (FGS). The use of FGS is growing in popularity (33) due to its cheap production, long shelf life, and sweeter taste (34). Since SSBs are one of the primary contributors to increased fructose intake (35), monitoring the amount of this sugar in SSB products is crucial for investigating dietary fructose intake and its related health outcomes.

Given the challenges described above, our study sought to verify whether the labeled sugar content in SSBs corresponds to the actual sugar content contained in these products, and if so, how the information can be utilized to enable the reliable compilation of branded food databases for use in national food monitoring studies. A secondary objective of our study was to determine the specific types of sugars found in SSBs in the Slovenian food supply, particularly the proportion of fructose.

## Materials and Methods

### Sample Selection

Our sample included the most consumed SSBs in Slovenia, which were selected using a market share approach. The selection of these beverages was thus based on the yearly nationwide (Slovenia) sales data provided by the NielsenIQ agency. The sales data were provided in Microsoft™ Excel worksheets in the universal form, which included barcode number, product name, and quantity of products sold from the year 2019. Information was available for the following selected NielsenIQ food categories: energy drinks, fruit juices, iced tea, mineral water, syrup, and soft drinks. In the next step, products contributing to 95% of the nationwide volume sales (N = 380) were re-categorized to tease out the different types of SSBs, i.e., sugar-sweetened colas, iced-tea drinks, sugar-sweetened energy drinks, flavored waters, and others, such as fruit and other carbonated drinks. In each of these subcategories, we summed the quantities of the same type of products sold, differing only in the package quantity/form. For example, sugar-sweetened Coca-Cola, which was available in plastic bottles (0.5; 1; 1.5; 1.75, 2 L), cans (0.25; 0.33 L), and glass bottles (0.25 L), was assigned to the same SSB type. From each of the subcategories, we then selected the top six SSBs sold. This sequential approach helped assure that the most representative products were sampled from each subcategory. Additional samples were also selected in descending order regardless of subcategory, to reach the total laboratory analysis capacity; this was capped at 51 analyses. The final sample consisted of 7 sugar-sweetened colas, 7 iced-tea drinks, 7 sugar-sweetened energy drinks, 10 flavored waters, and 20 other SSBs. Altogether the sample represented 69% of the national market share for the selected categories. The chosen SSBs were purchased from different retailers located in Ljubljana, Slovenia, in 2020 for laboratory analyses.

### Labeled Composition and Sugar Content

To provide insights into the feasibility of the market share-driven sampling approach, we collected data on the labeled sugar content for the SSBs in the original NielsenIQ dataset. Using the 2019 edition of the Slovenian branded foods database (4), which was compiled using labeled nutrition declarations, we were able to ascertain the labeled sugar contents for 309 SSBs linked to available yearly sales data.

For the selected products, the data were extracted directly from the labels of the purchased beverages. The product labels were photographed and used to extract the barcode numbers, product names, and nutritional declarations, including total sugar, the ingredients list, package quantity, expiration date, and manufacturer. Sources of sugars were identified using the ingredient lists.

### Laboratory Determination of Sugars

Laboratory analyses were performed in the Biotechnical faculty (University of Ljubljana, Slovenia). We analyzed the presence of free fructose, free glucose, and sucrose, using high-performance liquid chromatography (HPLC). Analyses of each sample were replicated, and average values were used for further calculations. Glucose (anhydrous for biochemistry), fructose (for biochemistry, purity HPLC ≥99.0 %), and sulphuric acid were obtained from Merck, Darmstadt, Germany; analytical grade sucrose was obtained from Kemika, Zagreb, Croatia. A mixed standard solution of glucose, fructose, and sucrose was prepared with double-deionised water (Milli-Q, Millipore Corp., Milford, MA, USA) of 18.2 MΩ/cm resistivity in the range from 1 to 20 g/l. Peak identification was based on HPLC retention times as compared with the standards. Peak integration was performed with ChemStation software (revision B.04.03-SP2). Quantitation was based on the external standard method using seven-point calibration curves fitted by linear regression analysis with the Data Analysis Tools in Excel. Samples were centrifuged at 3,000 × g for 10 min, and the supernatant was filtered through a 0.45 μm Chromafil® RC membrane (Macherey-Nagel, Düren, Germany). The filtrate was appropriately diluted before direct injection into the HPLC.

The HPLC system used in this study (Agilent 1260 Infinity, Agilent Technologies, Germany) was equipped with a G1322A degasser, a G1312B binary pump, a G1367E Hip ALS autosampler with G1330B FC/ALS autosampler thermostat, a G1316A thermostated column compartment, and a G1362A refraction index detector (RID). A total of 20 μl of the sample was injected on a column Aminex HPX-87H (BioRad, Richmond, CA) with a length of 300 mm x 7.8 mm i.d. and a particle size of 9 μm. The analysis was performed at 35 °C with a flow rate of 0.6 ml/min using isocratic elution with 5 mM H_2_SO_4_ as a mobile phase.

### Data Processing and Analysis

Data were processed and analyzed using Microsoft Excel 2019 (Microsoft, Redmond, WA, USA) and R 2020 (R Core Team, Vienna, Austria). An assessment of the market share sampling approach was completed using the Wilcoxon rank-sum test. The average labeled total sugar content in our sample of beverages with the highest sales (*N* = 51) was compared with all beverages with available sales and composition data (*N* = 309). The level of significance was set at *p* < 0.05.

To assess the labeled vs. the analyzed total sugars, we first determined legislative boundaries for each product based on the EC regulatory guidance on tolerances (12). For drinks with a sugar content below 10 g/100 mL, the acceptable deviation was ±2 g/100 ml, and for drinks with a sugar content above 10 g/100 mL, the acceptable deviation was ±20 %. We calculated the discrepancy percentage between the labeled and the analyzed total sugar contents and compared it with the acceptable deviation. Descriptive statistics were used to describe medians, 25th/75th percentiles of labeled/analyzed total sugar content, and the difference between them. To verify whether labeled sugar content data can be used to research and monitor the food supply, we compared the labeled and analyzed sugar contents as two independent samples. As the data were not normally distributed, differences were investigated with Mood's median test with the significance level set at *p* < 0.05.

We further applied sales-weighing to examine the differences between our sample and all beverages and between labeled and analyzed total sugar contents. Sales-weighted average total sugar content was calculated based on the quantity of products sold per year (L) and their total sugar content.

To investigate the different types of sugars in SSBs, we estimated the percentage of fructose in the products. This was calculated based on the products' analyzed free fructose and fructose from sucrose (sucrose content divided by two).

## Results

Although our study only sampled a small proportion of available SSBs, we were able to compare labeled total sugar content in the original sample (*N* = 309) with that of the laboratory analysis sample (*N* = 51), employing a sampling strategy by market share. In doing so, we found no statistically significant differences (*p* < 0.05) in the average labeled sugar content between the two samples— both for SSBs in general (8.2 vs. 7.9 g/100 ml, respectively) and across selected beverage categories (Supplementary Table 1)—suggesting that the composition of the study sample was consistent with a majority of the national market share volume. Additionally, the sales-weighted average labeled sugar contents were the same for both samples (8.8 g/100 ml; Supplementary Table 1). We observed that, in general, the sales-weighted sugar content was higher than the non-sales-weighted average.

### Compliance of Labeled Sugar Content With Laboratory Results

**Figure 1:**
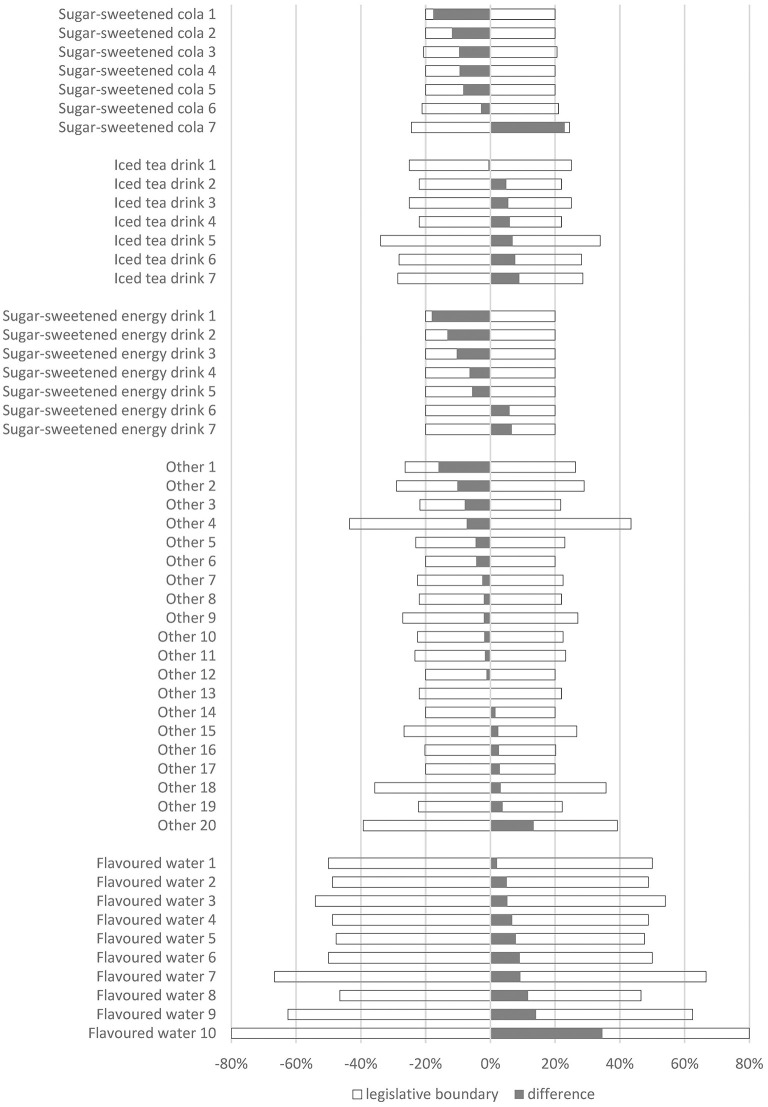
Difference (%) between labeled and analyzed total sugar content in sampled sugar-sweetened beverages and calculated EU tolerance ranges (%).

Compliance of the labeled sugar content with the analytically determined sugar content (as required by the EU regulatory tolerance range) for specific samples is shown in Figure 1. All 51 SSB samples were within the regulatory tolerance range of the labeled sugar content. The differences between the labeled and analytically determined sugar contents deviated almost equally each way, i.e., positively and negatively, with 24 drinks containing less sugar than labeled and 26 drinks containing more sugar than labeled. One sample contained the same amount of sugar as labeled. The observed difference range was from −2.0 g to +1.9 g per 100 mL (from −18% to 35%). Most samples (*N* = 41, 80%) had a deviation of ±10%. Only two sugar-sweetened colas and one energy drink had a deviation close to the regulatory limits. Interestingly, flavored waters always contained more sugar than labeled and also had higher deviations (2–35%). As a result of the lower sugar content in this subcategory, the regulatory relative tolerance ranges were also wider than for other soft drinks. A deviation in a positive direction was also observed in iced-tea drinks, where six out of seven beverages had higher analytically determined sugar than the labeled values. Moreover, sugar-sweetened colas (6 out of 7) and energy drinks (5 out of 7) contained less sugar than labeled.

Table 1 shows medians with 25th−75th percentile values for the analytically determined and labeled total sugar contents from the whole sample and the subcategories. The whole sample median for labeled and analyzed sugar contents was very similar (8.9 vs. 8.5 g/100 mL); the difference (−4%) was not statistically significant. Additionally, none of the five subcategories showed significant differences between labeled and analyzed total sugar contents. In absolute terms (g/100 mL), the largest differences in medians were observed in sugar-sweetened colas and energy drinks (−0.7 g). This coincided with the regulatory tolerance ranges for these SSBs. Drinks with a higher sugar content (>10 g/100 mL; sugar-sweetened colas and energy drinks) have a tolerance range defined by percentage (±20%), which allows for greater deviations in g/100 mL, while drinks with less sugar (<10 g/100 mL; flavored water) have a tolerance range defined by the amount (±2 g/100 mL), which allows for less deviations in g/100 mL.

**Table 1:** Labeled and analyzed values of total sugar content in different (sub)categories of sugar-sweetened beverages.

	***N***	**Labeled total sugar (g/100 mL)**	**Analyzed total sugar (g/100 mL)**	**Difference**		
		**Median (P25–P75)**	**Median (P25–P75)**	**g/100 mL**	**%**	***p***
Total	51	8.9 (5.6–10.1)	8.5 (5.8–9.9)	−0.4	−4%	ns
Flavored waters	10	4.0 (3.2–4.1)	4.2 (3.6–4.4)	0.2	5%	ns
Sugar-sweetened cola	7	10.3 (9.5–11.0)	9.6 (8.8–10.1)	−0.7	−7%	ns
Iced tea drinks	7	8.0 (7.0–9.1)	8.0 (7.6–9.6)	0	0%	ns
Sugar-sweetened energy drinks	7	11.0 (10.3–11.0)	10.3 (9.5–10.6)	−0.7	−6%	ns
Other sugar-sweetened beverages	20	8.9 (7.4–9.7)	8.6 (6.6–9.7)	−0.3	−3%	ns

*N, number of products; P25, 25th percentile; P75, 75th percentile; ns, not significant*.

### Types of Sugars in SSBs

Nutrition declarations on food labels only contain information concerning the total sugar content and not information about specific types of sugars. In Table 2, we present the results of the laboratory analysis of the contents of different types of sugars. In general, sampled beverages contained a similar amount of sucrose (median 22.2 g/L), free glucose (27.6 g/L), and free fructose (26 g/L), with notable differences between different subcategories. Sugar-sweetened colas contained the highest content of free glucose (42.1 g/L) and fructose (46.9 g/L), and lower amounts of sucrose (2.9 g/L). In contrast, flavored waters contained more sucrose (22.7 g/L), and less free glucose (9 g/L) and fructose (9.5 g/L). The overall median for the proportion of fructose (note: monosaccharides and fructose are present in sucrose) was 50% (range: 40–66%). An above-average proportion of fructose was found in sugar-sweetened colas (51%; range: 50–59%). Interestingly, sugar-sweetened energy drinks, which had the highest total sugar content, had the lowest proportion of fructose (42%; range: 40–50%).

**Table 2:** Analyzed values of sucrose, glucose, and fructose content in different (sub)categories of sugar-sweetened beverages.

	**Sucrose (g/L)**	**Glucose (g/L)^**a**^**	**Fructose (g/L)^**a**^**	**% Fructose^**b**^**
	**Median (P25–P75)**	**Median (P25–P75)**	**Median (P25–P75)**	**(min–max)**
Total	22.2 (10.2–38.3)	27.6 (14.1–37)	26 (14.5–36.5)	50 (40–66)
Other	16.9 (10.1–33.0)	28.2 (23.0–35.8)	33.1 (23.1–36.8)	50 (49–66)
Flavored waters	22.7 (17.9–29.4)	9.0 (6.6–10)	9.5 (7.1–10.1)	50 (49–61)
Sugar-sweetened cola	2.9 (0–3.8)	42.1 (41.3–49.7)	46.9 (42.6–51.1)	51 (50–59)
Iced tea drinks	40.8 (26.3–51.7)	27.6 (13.1–28.4)	24.9 (11.5–27)	49 (48–50)
Sugar-sweetened energy drinks	43.4 (23.3–52.6)	37.7 (26.4–43.4)	24.9 (20.2–28.4)	42 (40–50)

*P25, 25th percentile; P75, 75th percentile*.

a *Measured in monosaccharide form*.

b *Percentage (%) of fructose was calculated with consideration of free fructose and fructose present in sucrose*.

To generate further context and insights, these results were assessed by considering the sources of sugar, as provided in the ingredient lists (labeling information). Sucrose was found to be a key ingredient in the sweetening of selected SSBs (*N* = 36; 71%). We found four drinks with labeled fructose-glucose syrup (FGS) and seven with glucose-fructose syrup (GFS). FGS appeared in one sugar-sweetened cola and in other (carbonated) soft drinks (*N* = 3). Among the products with a high fructose content/proportion (defined by a minimum of 3 g total fructose per 100 mL and a proportion of fructose above 52%), there were two products with fruit juice and four products with FGS. Altogether, the proportion of fructose in FGS-containing beverages was between 59 and 61%. GFS was found in iced-tea drinks (*N* = 2) and other beverages (*N* = 5) containing fruit juice. We observed that the use of FGS or GFS is commonly associated with a specific manufacturer. When FGS or GFS appeared in one product, it was commonly used in other products from the same manufacturer. Interestingly, only one sugar-sweetened cola labeled the use of FGS, while others only listed the use of sucrose. Nevertheless, laboratory analysis showed high levels of free glucose and fructose in the cola subcategory. The use of FGS was not labeled in flavored waters and sugar-sweetened energy drinks. In addition to sucrose, most energy drinks (5 out of 7) also had glucose, or glucose syrup (GS) added, which tilted the fructose-to-glucose ratio in favor of glucose.

## Discussion

Studies comparing the labeled quantities of specific nutrients in food products with results from laboratory analyses are scarce. To the best of our knowledge, this is the first study to examine the sugar contents of beverages in the Slovenian food supply in this context. Additionally, the accuracy of nutrition labels is rarely the subject of concern for the authorities tasked with regulating food safety and quality. Food inspections, for example, are typically focused on food safety issues, microbiological and chemical safety, the presence of additives and undeclared allergens, etc. (15), rather than on the nutritional quality of food. As such, the responsibility for ensuring that the nutritional information on food labels is accurate lies with the manufacturers, who must follow regulatory tolerance ranges (12). Our study showed that the analyzed SSBs were generally within the EU tolerance ranges for sugars. This finding is similar to the results from a recent Spanish study (20) in which only 5% of SSBs exceeded the regulatory tolerance range, i.e., in all cases, the analytically determined sugar was below the labeled content. The Spanish study also reported similar results for other food categories, documenting an overall compliance rate with EU regulation of 98.4% (20). In Ireland, the results differed. O'Mahony et al. (19) reported that in yogurts, the sugar content was more likely to be non-compliant with the EC guidance, differing from the labeled value. Out of 200 sampled yogurts, 19% did not meet EU tolerance ranges, and significant differences were seen in all types of yogurts (natural, flavored, and luxury). Much better compliance, however, was reported for other nutrients, particularly for fats and saturated fats (3 and 5%, respectively). Albuquerque et al. investigated the compliance of mandatorily labeled nutrients in Portugal (18), but they focused primarily on fats, salt, and saturated fats; the observed compliance for these three nutrients was 88, 74, and 73%, respectively. Their study highlighted notable differences between different food categories, e.g., nutrient contents in fast food and potato products were typically overestimated, while they were generally underestimated in sauces. The reasons for the observed deviations from labeled values can be attributed to a variety of issues in the production process. For example, manufacturers commonly use calculations based on the food ingredients to estimate the nutrient content (11). These calculations frequently contain errors due to limited data on the nutritional composition of the ingredients; these values are then further confounded by the production process, batch-to-batch variability, and issues related to processing and product stability (18). Errors in calculations can also occur when a manufacturer employs laboratory analyses, e.g., problems related to inappropriate sampling and sample preparation (including homogenisation) or an inappropriate analytical method for the selected food matrix are potentially common occurrences (17).

Deviations, even within regulatory tolerance ranges, can also limit the reliability of the data in branded food databases. However, the results of our study did not show any significant differences between the labeled and analyzed sugar contents, either in the whole sample or among the specific subcategories, affirming previous findings concerning the sugar contents of SSBs and other food categories (20). While our results suggest that differences may occur when market share differences are considered, our sales-weighted figures should be taken with some caution since the beverage with the largest market share in Slovenia (cola-type drink) contains added FGS and can contain up to 5% of maltose and other sugars (36), and maltose and other sugars were not quantified in our laboratory analyses.

Our study results mostly indicate that the labeled sugar content information in SSBs is reliable for compiling branded food databases and for use in nutrition research. Branded food databases contain a large amount of data based on food labeling information, which in turn (if accurate) can help the responsible authorities and researchers compile a more comprehensive picture of food intake in the population and monitor food reformulation progress. For example, in Slovenia, such data are collected as part of the Composition and Labeling Information System (CLAS) (4). SSBs are a particularly important category because they are subject to reformulation activities. Recent studies have shown that reformulation changes can happen quickly in this category (37–40). Sugar reduction and the use of non-caloric sweeteners represent another cause of significant differences in the compositions of non-alcoholic beverages, even within the same subcategories (41). As a result, in nutrition research, it has become increasingly important for researchers to accurately assess sugar intake without under- or overestimating the sugar content of food products. Finally, branded food databases can play a vital role in policy development. When such databases are compiled and connected in a cross-sectional manner and across different time points, they can be very useful for generating insights on the effectiveness of food reformulation initiatives (6). Similarly, when these databases are combined with sales and/or consumer habits data, they can be used to inform emerging national and local food policies (42, 43).

Historically, data on the composition of SSBs, as they relate to the content of specific types of sugars, are very limited in the scientific literature. The results of our study showed that, in most beverages, the content of total fructose is about 50 %, which is consistent with the labeled ingredients for these products (most beverages were mainly sweetened with disaccharide sucrose, which is composed of fructose and glucose). This is a different situation than that observed in the U.S., where FGS (also commonly known as high fructose corn syrup) almost completely replaced sugar in SSBs (44). Beverages in the U.S. contain higher amounts of fructose, around 55%, reflecting the use of a standard version of FGS containing 55% of fructose (36). In our study sample, products sweetened with FGS included a few carbonated drinks (*N* = 4) with fructose proportions between 59 and 61%. The use of FGS is less common in beverages in Europe than in the US; in Europe, GFS with 42% fructose is sold as a standard (45). In our study sample, GFS was present in seven beverages, mainly in fruit drinks, in which the GFS offset the higher fructose content from fruit juice so that the final proportion of fructose was again around 50%, which is comparable with SSBs sweetened with sucrose only. Higher fructose levels were only seen in beverages with either FGS or higher fruit juice contents. Beverages with a higher percentage of fruit and 100% fruit juice are often perceived as a healthier choice. However, as a result of their high levels of naturally occurring sugar (especially fructose), studies suggest that their metabolic effects are very similar to beverages with added sugars (46). Our study showed that sugar-sweetened colas mainly consist of free glucose and fructose, regardless of whether sucrose or FGS is used for sweetening. Similar findings were reported in other studies, in which researchers hypothesized the potential usage of unlabelled FGS (16, 47). In aqueous solutions, sucrose can be subject to natural hydrolysis, which occurs in acidic conditions over time (45). Birkhed reported that in SSBs, the majority of sucrose can be hydrolysed after 5 months of storage at room temperature (48). A recent case study reported that this process is much faster in cola drinks than in other carbonated fruit drinks (49). For this reason, the content of monosaccharides in any final products cannot be used as a reliable indicator of specific sweetening ingredient use in SSBs. Since cola drinks with sucrose labeled in our study sample contained around 50 % fructose, and inverted sugar is rarely used in soft drinks (45), we believe that sucrose hydrolysis most likely took place in these samples. Meanwhile, energy drinks had the lowest proportion of fructose (42%) due to the use of glucose or GS. Glucose is a common ingredient in sport and energy drinks since it provides more energy per unit of sweetness and allows faster use of input energy (45). Nevertheless, even with a lower proportion of fructose, energy drinks still contained the highest amount of total sugar from the SSB drinks analyzed in the study, and, consequently, the amount of fructose (g/100 ml) in these drinks was similar to, and sometimes even higher than, other SSBs (50).

SSBs contribute significantly to fructose intake, generally due to the high sugar content and FGS use. In the US, the use of FGS is increasing (33). In the EU, the use of both FGS and GFS in SSBs was restricted by the Common Agricultural Policy (CAP), which contained a quota that strictly limited the quantities in SSB manufacture (45). In 2017, this quota was removed, and manufacturers were allowed to add caloric sweeteners in SSBs during the following years. The EC, however, does not expect these policy changes to cause an increase in the use of FGS and/or GFS (51). Further monitoring of these policy changes and their effects is essential, as fructose intake has already increased greatly in recent decades (52). Various studies have found associations between high fructose intake and increased risks for major non-communicable chronic diseases, such as metabolic syndrome, heart disease, obesity, diabetes, and dementia (31, 53–55). Various studies also suggest that high fructose consumption can negatively impact less physically active people (56). However, information on the negative health effects of the current levels of fructose consumption in Europe remains limited (57). The EC encourages the monitoring of fructose and its intake for the aforementioned reasons and requires that manufacturers inform consumers about the type(s) of sugar (sucrose, FGS, GFS, etc.) that is/are contained in food products (58).

### Strengths and Limitations

A major strength of our study is that we were able to analyse the most relevant SSBs in the food supply using the market share sampling approach. Although we only analyzed 51 beverages, the sampled products represented 69% of the national market share volume. However, this sampling approach has an important limitation: it does not provide insights into niche products with a low market share. Another limitation of the approach is that the samples were purchased in 2020, based on 2019 market share information, i.e., yearly market share data were only available for the previous year. While food labeling data are commonly used in nutrition research, very few studies have investigated the concordance of food labeling data to actual product composition. In this regard, our study represents a major contribution to this field of research, with results that can be used to inform the future efforts of food control authorities and policymakers in Slovenia and across the EU. The tolerances used in our study corresponded to the official EC guidance on regulatory tolerances, which are required for the nutritional declaration on food labels. In certain cases (i.e., for nutrition and health claims), this guidance provides stricter criteria, but they were not applied, as the study sampling approach was not designed in such a way as to capture a sufficient number of products labeled with such claims. Finally, we should note that, in the laboratory analyses, we only investigated the content of sucrose, glucose, and fructose, not other sugars. Other sugars may have been present in smaller quantities, and they could have affected the overall sugar content, sales-weighting, and proportion of fructose, especially in products with FGS, where maltose and other saccharides can represent up to 5% of the sugar content (36).

## Conclusions

Our study showed that the labeled sugar content in SSBs in Slovenia corresponded to the actual sugar content found in the food products, suggesting the usefulness and reliability of nutrition label information for compiling branded food databases and monitoring the food supply. Our case study also provided an example of how market share-driven sampling can be used for these types of verification studies. The results from the laboratory analyses and the assessment of the ingredients in SSBs showed that sucrose was the main sweetening component and that the proportion of fructose was typically around 50% across the sampled products. Given the possible changes in the use of caloric sweeteners, further monitoring of this area for food policy and nutrition research purposes is recommended, with a particular focus on FGS usage.

## Data Availability Statement

The datasets presented in this article are not readily available because restrictions apply. The raw data supporting the conclusions of this article will be made available by the authors without undue reservation (without disclosure of specific brands). Sales data were obtained for internal use only and can be ordered directly from NielsenIQ agency. Requests to access the datasets should be directed to Igor Pravst (igor.pravst@nutris.org).

## Author Contributions

IP: conceptualization. ŽL, MH, and SK: sample collection. MS: laboratory analyses. EH: data analyses, formal analysis, and writing—original draft preparation. IP and EH: methodology. IP, AK, KŽ, MS, and NP: manuscript writing—review and editing. All authors have read and agreed to the published version of the manuscript.

## Funding

Data collection for this study was supported by the national research programmes Nutrition and Public Health (P3-0395, funded by the Slovenian Research Agency) and Biochemical and biophysical characterization of natural compounds (P4-0121, funded by the Slovenian Research Agency), Infrastructure programme for monitoring of the composition and labeling of foods (IO-0054, funded by the Slovenian Research Agency), and research project L3-9290, funded by the Ministry of Health of Republic of Slovenia and Slovenian Research Agency. The study was conducted within the Food Nutrition Security Cloud project (FNS-Cloud), which received funding from the European Union's Horizon 2020 Research and Innovation Programme (H2020-EU.3.2.2.3.—A sustainable and competitive agri-food industry) under grant agreement no. 863059. Neither European Union institutions and bodies nor any person acting on their behalf may be held responsible for the use of the information contained herein. The funders had no role in the design of the study, in the collection, analyses or interpretation of data; in the writing of the manuscript, or in the decision to publish the results.

## Author Disclaimer

Information and views in this report do not necessarily reflect the official opinion or position of the European Union.

## Conflict of Interest

IP has led and participated in various other research projects in the area of nutrition, public health, and food technology, which were (co)funded by the Slovenian Research Agency, Ministry of Health of the Republic of Slovenia, the Ministry of Agriculture, Forestry, and Food of the Republic of Slovenia, and, in the case of specific applied research projects, also by food businesses. The remaining authors declare that the research was conducted in the absence of any commercial or financial relationships that could be construed as a potential conflict of interest.

## Publisher's Note

All claims expressed in this article are solely those of the authors and do not necessarily represent those of their affiliated organizations, or those of the publisher, the editors and the reviewers. Any product that may be evaluated in this article, or claim that may be made by its manufacturer, is not guaranteed or endorsed by the publisher.
