# Supplementary material for: Verifying the Use of Food Labeling Data for Compiling Branded Food Databases: A Case Study of Sugars in Beverages
Source: Front Nutr. 2022 Feb 3;9:794468. doi: 10.3389/fnut.2022.794468 (PMC8850951; doi:10.3389/fnut.2022.794468)
Supplement: Supplementary file 1 [file Table_1.docx]

Supplementary Material

**Supplementary Table 1:** Labelled and analysed average total sugar content in sugar-sweetened beverages.

|  | **Full sample^1^** | | | **Study sample^2^** | | | | | |
| --- | --- | --- | --- | --- | --- | --- | --- | --- | --- |
|  |  | **Labelled total sugar** | |  | **Labelled total sugar** | | **Analysed total sugar** | | |
|  | N | Average (g/100 ml) | Sales-weighted average  (g/ 100 ml) | N | Average (g/100 ml) | Sales-weighted average  (g/ 100 ml) | | Average (g/100 ml) | Sales-weighted average  (g/ 100 ml) |
| **Total** | **309** | **8.2** | **8.8** | **51** | **7.9** | **8.8** | **7.8** | | **8.4** |
| Flavoured waters | 34 | 3.5 | 3.6 | 10 | 3.7 | 3.9 | 4.0 | | 4.2 |
| Sugar-sweetened cola | 32 | 10.3 | 10.8 | 7 | 10.1 | 10.8 | 9.4 | | 9.5 |
| Iced-tea drinks | 33 | 7.3 | 8.2 | 7 | 7.7 | 8.7 | 8.2 | | 9.2 |
| Sugar-sweetened energy drinks | 42 | 9.3 | 10.0 | 7 | 10.8 | 10.7 | 10.1 | | 10.2 |
| Other sugar-sweetened beverages | 168 | 8.7 | 9.2 | 20 | 8.4 | 8.5 | 8.3 | | 8.4 |

Notes: ^1^All non-alcoholic beverages in selected categories in Slovenia with available sales and composition data; ^2^Selected beverages in our study, which represent 69% of national volume sales market.
